# Association between leisure noise exposure and hearing status in young Croatian adults

**DOI:** 10.2478/aiht-2025-76-3968

**Published:** 2025-09-30

**Authors:** Selma Cvijetić, Marija Kujundžić, Zrinka Franić, Mislav Malić, Davor Šušković, Siniša Fajt, Jelena Macan

**Affiliations:** Institute for Medical Research and Occupational Health, Division of Occupational and Environmental Health, Zagreb, Croatia; University Hospital Centre Zagreb, Department of Otolaryngology, Head and Neck Surgery, Zagreb, Croatia; Bontech Co., Zagreb, Croatia; University of Zagreb Faculty of Electrical Engineering and Computing, Department of Electroacoustics, Zagreb, Croatia

**Keywords:** cross-sectional study, headphones, hearing loss, recreational listening to music, speakers, gubitak sluha, presječno istraživanje, rekreativno slušanje glazbe, slušalice, zvučnici

## Abstract

Young people often use headphones or speakers and most visit noisy places recreationally. The aim of this cross-sectional study was to determine the relationship between exposure to recreational noise and hearing in 108 young Croatian participants aged 18–28 years. Hearing was assessed with audiometry and noise exposure by measuring the headphone volume to which each participant was accustomed while listening to music. Data on the daily use of headphones/speakers, visits to recreational noisy places, self-assessment of hearing, and health and demographics data were obtained with a questionnaire developed for this purpose. Mild sensorineural hearing loss was found in 5.5 % of participants (one man and six women). While all men reported their hearing as good, 13.9 % of women (n=9) described their hearing as average. Those women had significantly higher hearing thresholds than women who rated their hearing as good (p=0.036). Men spent significantly more time using headphones/speakers than women (1.9±1.2 vs 1.3±0.8 hours, p=0.002). Both men and women spent similar amount of time in recreational noisy venues, averaging 12.0 hours per month. More men used headphones at volumes exceeding 70 dB than women (52.2 % vs 27.7 %, p=0.009). Participants who self-reported hearing loss had significantly higher hearing thresholds than those who did not (p=0.036). Although we found no clear link between recreational noise and hearing loss, elevated thresholds in participants who reported hearing difficulties highlight the need for targeted hearing loss prevention.

## Abstract (hr)

Mladi ljudi često u rekreativne svrhe koriste slušalice i zvučnike, a većina njih posjećuje i bučna mjesta. Cilj istraživanja bio je utvrditi odnos između izloženosti rekreativnoj buci i kvalitete sluha u mladoj populaciji. U presječnom istraživanju sluh je procijenjen audiometrijom, a izmjerena je i uobičajena glasnoća u slušalicama. Podatci o svakodnevnoj upotrebi slušalica, posjetima bučnim rekreativnim mjestima, samoprocjeni sluha i zdravlju te demografski podatci prikupljeni su putem upitnika na 108 mladih sudionika u dobi od 18 do 28 godina. Blaga senzorineuralna oštećenja sluha pronađena su u 5,5 % sudionika (jedan muškarac i šest žena). Svi su muškarci subjektivno procijenili uredan sluh, a 13,9 % žena (n=9) opisalo je svoj sluh kao prosječan. Te su žene istovremeno imale značajno veće pragove čujnosti u usporedbi s onima koje su ocijenile svoj sluh urednim (p=0,036). Muškarci su svakodnevno rabili slušalice značajno dulje od žena (1,9±1,2 prema 1,3±0,8 h, p=0,002). I muškarci i žene provodili su sličan broj sati na rekreativnim bučnim mjestima, u prosjeku 12,0 sati mjesečno. Veći postotak muškaraca koristio je slušalice na glasnoći većoj od 70 dB u odnosu na žene (52,2 % prema 27,7 %, p=0,009). Sudionici koji su izvijestili o subjektivnom oštećenju sluha imali su značajno veći prag čujnosti (p=0.004). U naših ispitanika, muškarci su bili više izloženi rekreativnoj buci nego žene, i time izloženi većem riziku od gubitka sluha. Iako nije potvrđena povezanost između rekreativne buke i oštećenja sluha, ispitanici sa subjektivnim tegobama imali su veći prag čujnosti, što upućuje na potrebu za praćenjem i ciljanom prevencijom radi očuvanja sluha.

Exposure to noise affects people’s health directly and indirectly, as it can cause hearing impairment and diminish work ability and the quality of communication, concentration, and sleep (1, 2). While sensitivity to noise is individual and depends on the level and length of exposure, the effects of noise on hearing can range from mild and transient disturbances to permanent damage. Exposure to environmental noise originates from various sources. Recreational or leisure noise, according to the World Health Organization (WHO) (3), refers to all noise sources that people are exposed to through leisure activities, such as attending nightclubs, pubs, live sporting events, concerts or other music venues, and listening to loud music through headphones or speakers. For occupational and non-occupational noise exposure, the WHO recommends the 24-hour equivalent of continuous exposure level (LEQ) of 70 dBA, with a time-intensity exchange rate of 3 dB, which equals 80 dB for a maximum of 40 h a week, since higher levels are associated with adverse health effects (4, 5).

The risk from leisure noise exposure primarily concerns young people, who are increasingly exposed to noise through headphones or speakers or by attending loud places. Young people, including adolescents, often voluntarily expose themselves to high sound volumes during leisure. Nearly half the teenagers and young adults aged 12–35 years in middle- and high-income countries listen to loud music, and nearly 40 % are exposed to potentially damaging sound levels in nightclubs, discotheques, and bars (6). Relatively recent research indicates that between 60 % and 75 % of young people experience transitory tinnitus after leaving nightclubs, and as many as 18.3 % experience permanent tinnitus (7, 8). Only 3–10 % of young people report using ear protection in loud venues, even though most are aware of the harmful effects of noise on hearing (9, 10). Permanent effects can appear several years after frequent exposure to noise (11).

As far as volume recommendations for headphones or speakers are concerned, exposure should not exceed 1.6 Pa^2^h per week, a value derived from the WHO standard of 80 dBA for 40 hours per week (6). A recent review article (12) shows that the prevalence of exposure to excessive noise from headphones/speakers of 23.81 % is similar across age groups. Two other papers (13, 14) reveal that preferred listening volumes usually increase in noisy environments or when an earphone does not effectively buffer background noise. One systematic review (15) shows that up to 58.2 % of adolescents and young adults exceed the allowed daily noise limit, particularly if there is background noise.

As for the connection between recreational noise and hearing impairment in young people, findings vary: some studies report clear evidence of hearing impairment, others point only to subjective symptoms, while some find no association at all (16,17,18,19). However, the majority confirm a clear risk of hearing damage later in life, as regular or prolonged exposure to leisure noise can permanently damage the sensory cells of the inner ear and result in irreversible hearing loss (20). Considering that the early symptom is the inability to hear the high-frequency range, hearing impairment may not be obvious immediately, especially in young people. The aim of our study was therefore to 1) assess exposure to leisure noise in young people through volume measurements in headphones/speakers and through self-reported frequency of visiting loud places and 2) to determine the association between so determined exposure and subjective and objective hearing loss parameters.

## PARTICIPANTS AND METHODS

### Participants

Our study included 108 young adults (≥18 years of age), recruited among Zagreb University, Fire Academy, and high-school students. The study was conducted during the first semester of the academic years 2022/2023 and 2023/2024. Before inclusion, the candidates were informed about the research through lectures and written materials. Participation was voluntary and all participants signed an informed consent before inclusion. The exclusion criteria were chronic ear diseases or a previous acute disease that resulted in known hearing loss.

The study was approved by the Ethics Committee of the Institute for Medical Research and Occupational Medicine (IMROH), Zagreb, Croatia (document no. 100-21/22-08).

### Ear examination

Ear examination was conducted by a medical doctor at IMROH to assess the condition of the external auditory canal, tympanic membrane, and the middle ear. Prior to examination, we collected the history of past ear diseases, other diseases, and current medication.

### Audiometry

Pure-tone audiometry was performed by an experienced technician with a diagnostic Bell audiometer (Inventis s.r.l., Padova, Italy) in the audiometric chamber PRO45S-Maxi (Puma s.r.l., Settimo Milanese, Italy). The audiometer played tones in the frequency range of 125–8000 Hz with the starting power of 10 dB. Audiometric threshold was obtained for both ears as a pure-tone average for 500, 1000, 2000, and 4000 Hz. Audiograms were interpreted by an otolaryngologist subspecialised in audiology. Hearing loss of 21–40 dB on the audiogram was classified as a mild sensorineural hearing loss, according to the WHO classification (21).

### Headphones volume measurement

The usual headphones volume for each participant was measured with the Audioscan Verifit Model VF-1 Real-Ear Hearing Aid Analyzer 2325 (Etymonic Design Inc., Dorchester, Canada). After system calibration, the measurement was taken with a microphone probe placed a few millimetres from the tympanic membrane by inserting a silicone tube into the ear canal with the in-ear headphones. Music was played with a Xiaomi Redmi Note 11 smartphone (Xiaomi Corporation, Beijing, China), and the volume was adjusted to the individual level preferred by each participant when listening to music.

The measurement, which took place in a typical office room with no background noise, lasted 10 s and involved no discomfort or health risk for the participants. The results are expressed in dB.

In order to determine whether the participants were exposed to potentially damaging noise through headphones, we set the noise WHO threshold of 80 dB for a maximum of 40 h a week (4). If someone habitually used headphones at a volume above this threshold, their noise exposure was considered potentially harmful. The hours a week information we collected in the questionnaire described below.

### Questionnaire

To evaluate subjective perception of noise-induced hearing loss in our participants, we developed a 32-item self-reporting questionnaire based on the 28-item Massachusetts Eye and Ear Infirmary questionnaire (Boston, MA, USA) described elsewhere (22, 23). Our questionnaire included information on basic demographics (age, gender, educational level), general health status, hearing problem symptoms, headphones/speakers usage habits, noise exposure through headphones, frequency of visiting loud places, subjective assessment of hearing, subjective assessment of headphones/speakers volume, and opinion on impact of noise on hearing. All 108 participants completed the questionnaire.

### Statistics

Data are shown as means ± standard deviations for continuous variables and as number and percentage for categorical variables. The distribution of variables was tested with the Kolmogorov-Smirnov test. Since most variables were not normally distributed, we applied the Mann-Whitney *U* test for differences between continuous variables and the chi-squared test for categorical variables. The relationship between any two, either continuous or categorical, variables was tested with Spearman’s correlation.

From questionnaire responses we derived total daily time of using headphones/speakers (h/day). Similarly, data on visiting noisy places for entertainment (night clubs, concerts, cinema) are expressed as total time per month (h/month).

Data were analysed using the Statistica software, version 15.0 (StatSoft Inc., Tulsa, OK, USA). P value lower than 0.05 was considered significant.

## RESULTS

Table 1 shows participant demographics and health data. Young men and women differed significantly in age and education but not in medical history or otoscopy findings. Otoscopic examination revealed that only 25.1 % of participants had cerumen in one or both ears. The most common reported chronic disease was an allergy.

**Table 1:** Demographic and health characteristics of the participants

	**Mean ± SD or N (%)**		p*
	**Men (N=43)**	**Women (N=65)**	
Age (yrs.)	22.0±2.5	23.1±1.7	**0.004**
Educational status:			
- university students	14 (32.6)	62 (95.4)	**<0.001**
- high-school students	6 (13.9)	2 (3.1)	**<0.001**
- fire academy	23 (53.5)	1 (1.5)	**<0.001**
Previous otitis	7 (16.3)	12 (18.5)	0.735
Chronic diseases	8 (18.6)	17 (26.1)	0.335
Medicine taking	5 (11.6)	10 (15.4)	0.541
Otoscopy (cerumen, infections, abnormalities)	10 (23.2)	18 (27.6)	0.565

* Mann-Whitney test or chi-squared test

We also found no gender differences in the hearing status of our participants (Table 2). Around half reported having hearing symptoms such as tinnitus, pain in the ear, and sensitivity to noise. Subjective perception of slightly impaired hearing (self-classified as *average*) was reported by nine women (13.9 %) and no man. Audiometry revealed mild sensorineural hearing loss in either right or left ear in one man (2.3 %) and five women (7.7 %), making up 5.5 % of all participants. The mean hearing threshold in both ears in both groups ranged between 11.2 and 13.5 dB.

**Table 2:** Hearing status of the participants

	**Mean ± SD or N (%)**		p*
	**Men (N=43)**	**Women (N=65)**	
Subjective symptom – single (tinnitus, pain or sensitivity to noise)	20 (46.5)	38 (58.4)	0.182
Subjective symptoms (multiple)	3 (6.9)	13 (20.0)	0.560
Self-assessment of hearing:			
- good	43 (97.7)	56 (86.1)	0.219
- average	0	9 (13.9)	0.225
- bad	0	0	/
Hearing threshold, right ear (dB) †	11.2±2.5	12.6±3.7	0.089
Hearing threshold, left ear (dB) †	11.2±1.7	12.1±3.7	0.809
Hearing loss (mild sensorineural)	1(2.2)	6 (9.2)	0.142

* Mann-Whitney test or chi-squared test.

† Mean at 500, 1000, 2000, 4000 Hz

However, gender differences came to the fore in headphones/speakers use (Table 3), most notably in the total use time a day, especially in gaming. More women reported to prefer headphones, especially the in-ear type, but did not differ from men in the subjective perception of high headphones/speakers volume (self-reported as *cannot hear background traffic and conversation*) while listening to music or gaming. However, the average measured headphones volume (dB) was significantly higher in men than in women, exceeding the >70 dB continuous exposure limit in 27.7 % of women and 52.2 % of men (p=0.009). Given that the average time of headphones/speakers use was 1.9±1.2 h in men and 1.3±0.8 h in women, our participants were still below the WHO risk threshold of developing hearing loss, yet the majority self-assessed some level of hearing loss risk (Table 4). Most women (61.4 %) assessed low risk, while men equally (37.2 %) assessed medium and low risk. Self-reported time spent in recreational noisy venues (loud places) did not differ between genders (12.8±8.0 h/month for men vs 11.3±7.4 h/month for women).

**Table 3:** Headphones/speakers usage in participants

	**Mean ± SD or N (%)**		p*
	**Men (N=43)**	**Women (N=65)**	
Listening music on weekdays (h/day)	2.3±2.3	2.2±1.6	0.711
Listening music on weekends (h/day)	3.3±2.0	2.7±1.9	0.133
Gaming on weekdays (h/day)	0.7±1.2	0.1±0.4	**0.003**
Gaming on weekends (h/day)	1.5±2.0	0.2±0.5	**<0.001**
Total time of using headphones/speakers (h/day)	1.9±1.2	1.3±0.8	**0.002**
Headphones	11 (25.6)	36 (55.3)	**0.002**
Speakers	9 (20.9)	5 (7.7)	**0.039**
Both	23 (53.5)	24 (36.9)	0.113
Type of headphones			
- in-ear	25 (58.1)	59 (90.8)	**<0.001**
- over-ear	6 (13.9	3 (4.6)	0.093
- both	12 (27.9)	3 (4.6)	**0.001**
Self-reported listening at high volume (*cannot hear background traffic and conversation*)	17 (39.5)	17 (26.1)	0.250
Measured headphone volume (dB)	70.3±10.0	65.5±9.6	**0.039**

* Mann-Whitney *U* test or chi-squared test

**Table 4:** Time spent at noisy places and self-assessment of hearing loss

	**Mean ± SD or N (%)**		p*
	**Men (N=43)**	**Women (N=65)**	
Visiting noisy places (h/month)			
- Night club	10.0±5.9	8.4±5.3	0.356
- Concert	9.2±5.0	7.6±2.7	0.320
- Cinema	4.1±3.3	5.2±2.4	0.243
Total time of visiting noisy places (h/month)	12.8±8.0	11.3±7.4	0.320
Self-assessment of hearing loss risk			
- High risk	2 (4.6)	1 (1.5)	0.344
- Medium risk	16 (37.2)	12 (18.4)	**0.035**
- Low risk	16 (37.2)	40 (61.4)	**0.010**
- No risk	9 (20.9)	10 (15.3)	0.490

* Mann-Whitney test or chi-squared test

We did not find any significant correlation between the hearing threshold and objectively measured headphones volume, total daily time of using headphones/speakers or time spent at loud places. We also did not find any significant difference in the hearing threshold between participants who listened to music with headphones/speakers at volumes above and below the 70 dB limit.

Participants who reported to use headphones/speakers at higher volumes to cancel background noise (*cannot hear background traffic or conversation*) also used headphones/speakers significantly more often than those who did not report increasing the headphones/speakers volume to cancel background noise (p=0.004). Furthermore, participants who reported exposure to higher volume also had significantly higher headphone volume measured by Verifit than those who reported listening to music at lower volumes (p<0.001). Participants who assessed their hearing as *average* had significantly higher hearing threshold than those who assessed their hearing as *good* (13.1 vs.11.7 dB) (p=0.036).

## DISCUSSION

In our young population aged 18–28 years, the prevalence of mild sensorineural hearing loss was 5.5 %. Although there are numerous studies on hearing impairment in young people, many do not show prevalence. In the United States, the overall prevalence of hearing loss among 6–19-year-olds, exceeding 15 dB in either ear, was 11.24 % in the past decade (24). In Slovakia, audiometry testing in 41 young university students showed a shift in hearing threshold at higher frequencies (8000 Hz) in 22 % of participants (17). In Poland, a small study in 58 young adults (19) reported noise-induced hearing loss in 6.9 % and another (25) in 4.2 %, this time in a much larger sample of 230 students.

However, most of our participants (75 %) with objectively measured hearing impairment reported no subjective experience of hearing loss, which suggests that young adults hardly notice mild hearing impairments, as they do not significantly impair their quality of life.

Those who reported listening to music at louder volumes to cancel background noise (questionnaire item: *cannot hear the background traffic and conversation*) also reported to use headphones/speakers significantly more often than those who reported applying lower volumes. This indicates that young people who practice unsafe use of headphones/speakers and do not have objective hearing impairment may still run a significant risk for later hearing loss development.

Our participants who reported a slight hearing loss (classified as *average hearing*) also had a higher hearing threshold (indicative of loss) than those who reported good hearing. This suggests that the subjective feeling of hearing impairment may predict its actual development later in life. People who subjectively feel that their hearing is impaired are probably more prone to subtle cochlear damage and may experience hearing problems undetected by standard audiometry (26, 27).

Although we did not find a correlation between time spent in noisy places and either subjective or objective hearing loss, a number of studies (7, 28,29,30,31) warn of transient hearing loss and tinnitus immediately after exposure in such venues and the risk of later permanent damage.

Average time spent on recreational use of headphones in all our participants, either for listening to music or gaming, was 1.5±1.1 h/day, which is similar to some other studies reporting up to two hours of use per day (16, 25). Mean values of sound pressure in the headphones of 66.5 dB in our participants did not exceed the recommended exposure levels of 70.0 dB, although the values in men were borderline. Such exposure is well below the high risk threshold of >90 dB for headphone volumes indicated by most studies (13, 32).

Our results also showed no significant correlation between headphone volume and the shift in the hearing threshold as indicator of hearing impairment. This is in line with some studies (33), including the recent review of 20 articles (34) that find no clear relationship between recreational noise activities and harmful effects on hearing (33, 34). However, other studies clearly evidence the negative effects of headphones/speakers on the auditory system (16,17,18). One interesting finding, published by Pawlaczyk-Łuszczyńska et al. (19), was worse hearing in irregular headphones users than the regular ones. All those discrepancies in findings might be owed to different methods of measuring exposure and outcome between studies.

Gender differences in listening volume found in our participants confirmed reports of several other studies, and it seems that men prefer louder music than women (32, 35, 36). However, we found no significant gender differences in objective hearing parameters, which is also in line with some earlier reports (36,37,38).

The interpretation of our findings is somewhat limited due to a relatively small sample and cross-sectional design, which does not allow establishing a definitive association between hearing loss and exposure to recreational noise. However, our objective audiometric assessment of hearing and objective measure of sound volume in the headphones are reliable enough to draw some conclusions.

## CONCLUSION

The advantage of our study is that it relies on objective assessment, including audiometry and direct measurement of listening volume through headphones/speakers. It also puts together data on recreational exposure to noise through headphones and visits to loud recreational venues. Although most young people in our study are not exposed to excessive noise on average, the findings still point to long-term risks of noise-induced hearing loss, given that one-third perceive themselves to be at moderate or high risk of developing hearing impairment.

Another curious finding are differences in listening habits between genders, most notably that young men expose themselves to higher levels of recreational noise. However, consequences of such behaviour are not apparent yet, as we have found no differences in objective hearing loss distribution between the genders.
